# Abomasal displacement in neonatal dairy calves: Review of recent literature with special emphasis on abomasal torsion

**DOI:** 10.14202/vetworld.2019.1121-1125

**Published:** 2019-07-25

**Authors:** Zuhair Bani Ismail, Faisal Omoush

**Affiliations:** Department of Veterinary Clinical Sciences, Faculty of Veterinary Medicine, Jordan University of Science and Technology, Irbid 22110, Jordan

**Keywords:** abdominal surgery, abomasal diseases, Holstein calves, roughage feeding

## Abstract

**Aim:**

This study aimed to describe the clinicopathological and necropsy findings in neonatal dairy calves affected with right torsion of the abomasum.

**Materials and Methods:**

The history and findings of clinical examination, hematology and serum biochemical analyses and necropsy were described in six neonatal Holstein calves with a confirmed diagnosis of right torsion of the abomasum. Furthermore, a review of the literature was carried out using internet search engines such as PubMed and Google Scholar concerning abomasal displacement in calves. Only published papers in scientific and refereed journals were reviewed.

**Results:**

Six neonatal Holstein calves (four females and two males) aged between 7 and 21 days were diagnosed with right torsion of the abomasum during necropsy. The calves were presented with peracute signs of anorexia, dehydration, abdominal pain, and abdominal distension. Hematology and serum biochemical analyses revealed hemoconcentration and azotemia, hyponatremia, hypochloremia, and hypokalemia.

**Conclusion:**

Abomasal torsion in neonatal calves must be placed on the deferential diagnostic list of calves suffering from peracute signs of abdominal pain and distension. Although the underlying etiopathological factors are not fully known, correction of nutritional mismanagement is required to prevent the condition.

## Introduction

Displacement of the abomasum is a common and multifactorial disease of primarily adult dairy cows [1,2]. The condition occurs mainly during peak milk production after parturition when cows are fed high concentrate and low roughage diets [1,2]. Usually affected cows suffer from multiple periparturient diseases along with displacement of the abomasum such as subclinical hypocalcemia, ketosis, dystocia, stillbirth, and metritis [1-6]. Other predisposing factors of the condition may include breed, age, and season [3,4]. Normally, the abomasum is full of liquid contents and lies on the right cranioventral floor of the abdomen [3-5]. Displacement usually occurs when the abomasum becomes dilated with gas leading to atony and migration to the left or right side of the abdomen [1-6].

In calves, displacement of the abomasum occurs only rarely [7-12]. Clinically, the condition is characterized by depression, partial or complete anorexia, dehydration, weight loss, abdominal distension, abdominal discomfort, chronic bloat, and sometimes diarrhea [7-12]. Diagnosis in calves is accomplished by abdominal auscultation and percussion, and detection of the distinctive ping sound over the 10^th^ to the 13^th^ intercostal spaces on the left or right side of the abdomen [7-12]. Confirmation of the diagnosis is usually achieved using ultrasonography or intraoperatively [7-12]. Right volvulus of the abomasum is extremely rare in young calves. The condition is characterized by sudden onset of acute abdominal pain, right side distension, dehydration, and rapid deterioration and death [10,11].

In this study, all published data in refereed scientific journals regarding displacement of the abomasum in dairy and beef calves are compiled and summarized. In addition, a comprehensive description of the clinical, hematological, serum biochemical changes, and necropsy findings in dairy calves affected with right side displacement of the abomasum is provided.

## Materials and Methods

### Ethical approval

No ethical approval was required to perform this study.

### Informed consent

Written permissions were obtained from the farm owner before this study was carried out.

### Case history and clinical examination

This study was performed using six Holstein calves affected naturally with right torsion of the abomasum. Affected calves were subjected to a complete physical examination including heart rate, respiration rate, rectal temperature, mucous membrane color and capillary refill time, rumen motility, and auscultation, and percussion of the left and right middle abdominal sides.

### Laboratory analyses

Whole blood was collected from affected calves through jugular venipuncture and placed in plain and ethylenediaminetetraacetic acid-containing blood collection vacutainer tubes. Blood samples were transferred to the laboratory for analysis within 4-6 h on ice. In the hematology analyses, the total white blood cell count, red blood cell count, hemoglobin concentration, packed cell volume (PCV), mean corpuscular volume, and mean corpuscular hemoglobin concentration were determined using an electronic cell counter (Scil Vet ABC Hematology Analyzer, Scil Animal Care Company, USA). In the serum biochemical analyses, the total protein (TP), fibrinogen, glucose, blood urea nitrogen (BUN), creatinine, calcium, potassium (K), sodium (Na), and chloride (Cl) were determined using previously published methods [6].

### Literature review

A comprehensive review of the literature using search engines such as Google Scholar and PubMed using keywords such as abomasal diseases in calves, displacement of the abomasum in calves, right/left displacement of the abomasum in calves, surgery of the gastrointestinal tract in calves, and gastrointestinal diseases in calves was used to extract all published data concerning displacement of the abomasum in both dairy and beef calves. Only papers published in international and refereed journals were selected.

## Results

### Case description

An unusual outbreak of right side displacement of the abomasum in dairy calves <1 month of age was diagnosed in one dairy farm located in Al-Dulaial region in Jordan. During 1 month period (February 2019), a total of six calves were affected. The calves (four females and two males) were 7-21 days of age with an average age of 15 days. The calves were born in 2000 lactating Holstein dairy farm. Calves were fed 4-6 L of good quality colostrum within the first 6 h of life. Calves were housed separately in cages with metallic slatted floor approximately 30 cm above the ground inside a well-ventilated barn. The calves were reportedly fed cow’s milk at 10% of their body weight twice a day. No antibiotics or any feed additives were added to the milk. A 20% crude protein, concentrate-based calf starter (Table-1) that was produced on the farm was provided to the calves *ad libitum* starting from day 3 of age. No roughage was offered to the calves. Fresh water was offered *ad libitum*.

**Table 1:** Composition of calf starter used on the farm.

Component	kg/1000 kg feed
Corn	320
Soybean meal (48% protein)	230
Barely	275
Wheat bran	150
Sodium chloride	5
Sodium bicarbonate	5
Yeast	5
Fungal antitoxin	2
Menials and vitamin premix	3
Dicalcalcium phosphate	5

### Clinical presentation and physical examination findings

Clinically, affected calves were reportedly normal at one feeding time after which abdominal discomfort was noticed by the farm caretakers. The condition of the calves deteriorated rapidly with severe right side abdominal distension. Physical examination by the farm resident veterinarian revealed tachycardia (heart rate 120-170 beats/min), tachypnea (respiration rate 50-80 breaths/min), subnormal rectal temperature (38-38.6°C), moderate to severe dehydration (8-10%), and prolonged capillary refill time (>4 s). Abdominal auscultation and percussion revealed distinctive ping sound on the mid-abdominal region indicative of right side abomasal displacement or torsion. Treatments that were attempted in the farm were all futile and included passing an oral-ruminal tube, trocharization of the distended viscus on the right side using 16 gauge hypodermic needle, and parenteral administration of mineral oil.

### Hematology and serum biochemistry analysis

In the hematology analysis, all values were within normal limits except a slight leukocytosis and increased PCV (Table-2). In the serum biochemistry analysis, there were increased serum concentrations of TP, fibrinogen, blood urea nitrogen, and creatinine while the concentrations of glucose, Na, Cl, and K were significantly reduced (Table-3).

**Table 2:** Hematology analysis of calves with right torsion of the abomasum.

Parameters	Affected calves	References range[13]
RBC (×10^6^ cells/ml)	14	5.1-7.6
Hb (g/dl)	10	8.5-12.2
PCV (%)	52	22-33
MCV (fl)	40	38-50
MCH (pg)	12	14-18
MCHC (%)	30	36-39
Platelets (×10^3^ cells/ml)	560	193-637
WBC (×10^3^cells/ml)	14	4.9-12.0
Neutrophils (%)	68	36.7-52.5
Eosinophils (%)	1	0-7.5
Basophils (%)	0.5	0-2.5
Lymphocytes (%)	27	29.4-47
Monocytes (%)	3.6	0-6.7

RBC=Red blood cell, Hb=Hemoglobin, PCV=Packed cell volume, MCV=Mean corpuscular volume, MCH=Mean corpuscular hemoglobin, MCHC=Mean corpuscular hemoglobin concentration, WBC=White blood cell

**Table 3:** Serum biochemistry analysis of calves with right torsion of the abomasum.

Parameters	Affected calves	References range[14]
Total protein (g/l)	90	5.67-7.14
Fibrinogen (mg/dl)	700	270-820
Blood urea nitrogen (mg/dl)	33	3.3-19.4
Creatinine (mg/dl)	2.5	0.58-1.03
Calcium (mg/dl)	8.3	9.5-11.2
Glucose (mg/dl)	33	42-104
Sodium (mmol/l)	113	136-146
Potassium (mmol/l)	3.0	3.4-4.9
Chloride (mmol/l)	84	94-104

### Necropsy findings

**Figure-1:**
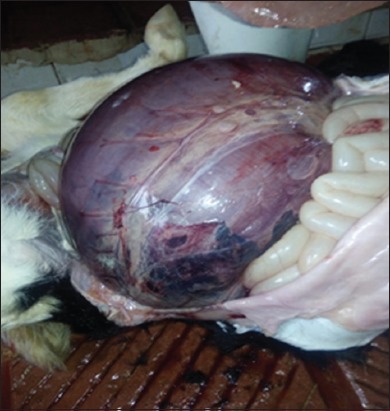
Right abomasal torsion in a neonatal dairy calf. The abomasum is severely distended with wall edema, congestion, and patches of hemorrhage.

**Figure-2:**
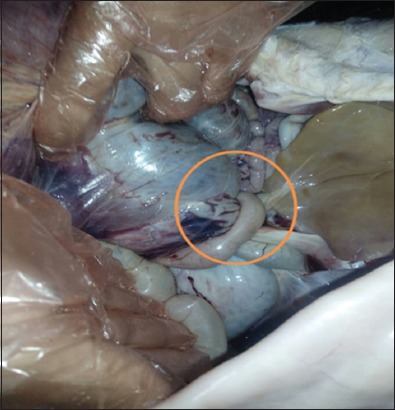
Right abomasal torsion in a neonatal dairy calf. The torsion was 360° counterclockwise at the pyloric region (orange circle).

**Figure-3:**
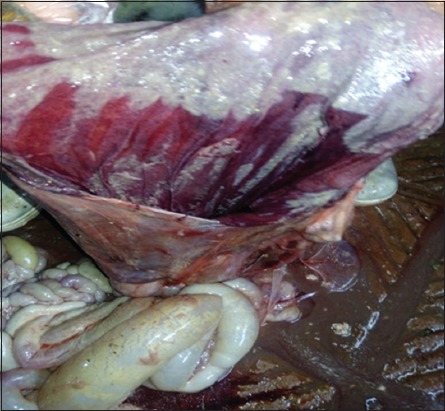
Right abomasal torsion in a neonatal dairy calf. Mucosal edema and congestion. No ulcers were found.

The affected calves were reportedly dead with 6-12 h after the onset of clinical signs. Necropsy examination was performed in all calves. The carcass appeared in poor body condition. Examination of the abdominal cavity revealed distended abomasum with gas and fluid. The wall of the abomasum was purple in color, edematous, and severely congested with patches of hemorrhage (Figure-1). Close examination of the abomasum revealed a 360° counterclockwise torsion at the pyloric region (Figure-2). The omasum was not involved in the torsion. No other abnormalities were found. By opening of the abomasum, the mucosa was congested and edematous with no discrete ulcerative lesions that could be seen (Figure-3).

### Literature review

Right side displacement of the abomasum in neonatal dairy calves is extremely rare. In fact, review of all literature concerning both right and left displacement of the abomasum in dairy and beef calves revealed only six published scientific papers since 1984 [7-12] (Table-4).

**Table 4:** Review of literature of published case reports of abomasal displacement in calves.

Diagnosis	Breed	Sex and age	Treatments	Outcome	References
Right displacement of the abomasum and abomasal ulceration	Holstein	8weeks old, female	Right flank abomasopexy	Recovered	[7]
Left displacement of the abomasum	Beef	6 months old, four males	Rolling, right paramedian abomasopexy	Favorable outcome in three calves out of four operated	[8]
Right displacement of the abomasum	Holstein	1year old, male	Medical treatment only	Unspecified	[9]
Right torsion of the abomasum and abomasal ulceration	Brown Swiss	45days old, male	Right flank abomasopexy	Recovered	[10]
zRight torsion of the abomasum	Holstein	1 month old, female	Right flank exploratory laparotomy	Euthanatized	[11]
Left displacement of the abomasum and bronchopneumonia	Holstein	8 months old, male	Rolling only	Slaughtered 2 months later	[12]

The clinical, ultrasonographic, and surgical findings of right displacement of the abomasum and abomasal ulceration were described in an 8-week-old female Holstein calf [7]. The diagnosis was confirmed by ultrasonography and during laparotomy. Treatment, in this case, was attempted using right-flank abomasopexy. Ulcerative lesions were detected in the mucosal surface of the abomasum. The calf was recovered uneventfully and returned to the herd following surgery [7].

Left displacement of the abomasum in four beef calves was reported, and the clinical presentation, diagnosis, and treatment were described [8]. The calves were <6 months of age. The calves suffered loss of appetite and left-sided abdominal distention. The diagnosis of left displacement of the abomasum was confirmed by ultrasonography. Non-surgical treatment was successful in all calves by rolling, but recurrence was diagnosed in three calves within 1 h to 6 days. Right paramedian abomasopexy was then performed in all cases with a successful outcome in only three of them [8].

In Holstein male calf, a diagnosis of right displacement of the abomasum was confirmed based on case history, clinical findings, and axillary diagnostic tests [9]. The calf was <1 year of age with a 5 days history of inappetence and constipation. In this case, tympanic and splashing sounds were heard over the middle third of the right side of the abdomen. Axillary tests were performed and included paracentesis of the tympanic area and rectal examination.

Right displacement of the abomasum complicated with abomasitis was diagnosed in a 45-day-old unweaned Brown Swiss male calf [10]. The condition was confirmed by ultrasonography and treatment was attempted surgically through right flank laparotomy approach. Abomasal wall edema and mucosal ulcerations were seen in this calf intraoperatively. The calf recovered uneventfully and was returned home after the operation [10].

The clinicopathological findings of right torsion of the abomasum in a 1-month-old Holstein heifer calf were presented [11]. The calf was reportedly presented with severe abdominal distention. The condition was diagnosed during right flank exploratory laparotomy during which a 360° counterclockwise torsion of the abomasum was confirmed. The animal was euthanatized intraoperatively because of severe vascular compromise of the abomasum and poor prognosis [11].

Chronic left displacement of the abomasum was diagnosed in an 8-month-old dairy Holstein male calf based on physical examination findings of ping sound on auscultation and percussion of the left side of the abdomen [12]. The calf was presented initially for respiratory problem evaluation. Correction of the abomasal displacement was achieved successfully by rolling initially, but recurrence was diagnosed 2 months later, and the calf was slaughtered because of poor growth [12].

## Discussion

Displacement of the abomasum is rarely diagnosed in calves. In this article, a comprehensive description of the clinical, clinicopathological, treatment options, outcome, and necropsy findings in six Holstein neonatal calves is provided. This outbreak occurred during the winter month of February in one dairy farm. In this farm, the calves are fed cow’s milk and supplemented with a concentrate-based, finely ground calf starter *ad libitum*. Analysis of this concentrate diet revealed approximately 20% crude protein. The diet was clearly deficient of dietary fiber as no roughage was offered to the calves. Although difficult to prove, we believe that this was the underlying predisposing factor in this outbreak. In adult dairy cows, displacement of the abomasum is usually associated with poor nutritional management during the transitional period [13-21]. As a result, negative energy balance is magnified in the immediate post-parturient period leading to various metabolic diseases including displacement of the abomasum [13-21]. These conditions are quite similar to what is being reported here in affected calves. Moreover, a recommendation to supplement the calves on this farm with good quality roughage was made and implemented, which resulted in the prevention of the problem.

The clinical progression of the condition in calves affected with the right torsion of the abomasum was rapid due to cardiovascular and respiratory collapse [10,11]. Usually affected calves with a right displacement of the abomasum are presented with acute abdominal signs such as colic, bilateral abdominal distension, respiratory distress, and dehydration [7,9-11]. Indeed, calves in this report died within 6-12 h after the beginning of clinical signs and were presented with a history of acute death following a short period of abdominal pain, distension, and dehydration. The hematology analyses showed hemoconcentration, hyperproteinemia, increased BUN, and creatinine which are normally indicative of dehydration. These findings are similar to those reported in adult cows with the same condition [13-21]. In addition, cows with abomasal torsion are usually presented with metabolic alkalosis early in the disease but later it can develop to metabolic acidosis [13-21]. Similarly, in this report, the calves here showed a significant degree of hyponatremia, hypochloremia, and hypokalemia which is most likely an indication of metabolic alkalosis. Furthermore, the serum glucose concentration was lower than normal in these calves which may indicate energy depletion due to anorexia and increased metabolic demands.

## Conclusion

Although rare, the literature review performed in this article and the case report of several calves affected with right torsion of the abomasum indicates that this condition could be encountered in practice in neonatal calves and must be placed on the differential list of calves suffering from anorexia, abdominal pain, and abdominal distension. The underlying etiopathological factors are probably similar to the condition in adult dairy cows. The condition progresses rapidly and immediately medical (intravenous fluid therapy and correction of acid-base imbalance) and surgical treatment to replace the displaced abomasum are essential for a positive outcome. Correction of underlying nutritional mismanagement is required to prevent the condition.

## Authors’ Contributions

ZBI: Manuscript writing and farm consultation; FO: Performed field work and primary case care. Both authors read and approved the final manuscript.
